# Gibberellic Acid (GA_3_): A Versatile Chiral Building Block for Syntheses of Pharmaceutical Agents

**DOI:** 10.1002/cbdv.202401823

**Published:** 2024-11-04

**Authors:** Zein Alabdeen Khdar, Tam Minh Le, Zsolt Szakonyi

**Affiliations:** ^1^ Institute of Pharmaceutical Chemistry University of Szeged Eötvös utca 6 H-6720 Szeged Hungary; ^2^ HUN-REN–SZTE Stereochemistry Research Group of the Hungarian Academy of Sciences Eötvös utca 6 H-6720 Szeged Hungary

**Keywords:** Gibberellins, Gibberellic acid (GA_3_), Stereoselective syntheses, Pharmaceutical agents, Building block

## Abstract

Gibberellic acid (GA_3_), an *ent*‐kaurene tetracyclic diterpene, has been considered to be a chiral pool for the chemical transformation of significant heterocyclic compounds. This chiral pool continues to influence modern synthetic chemistry as an inexpensive and versatile starting material since it is widely applied in agriculture. This review focuses on the stereoselective syntheses of bioactive agents with pharmaceutical potency prepared from Gibberellic acid.

## Graphical abstract

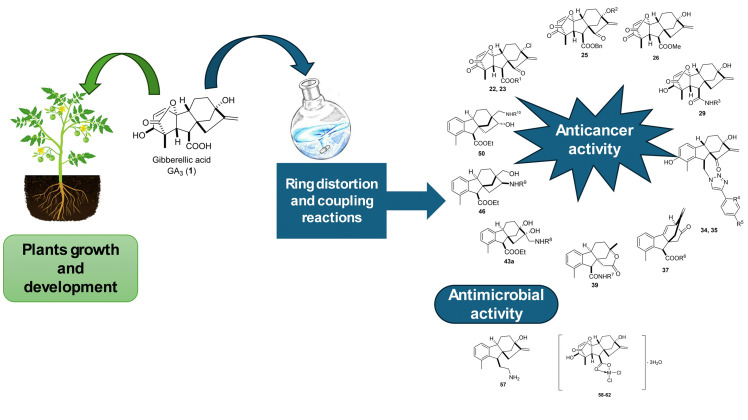

## 1 Introduction

### 1.1 Historical Perspective and Classification of GAs

**Figure 1:**
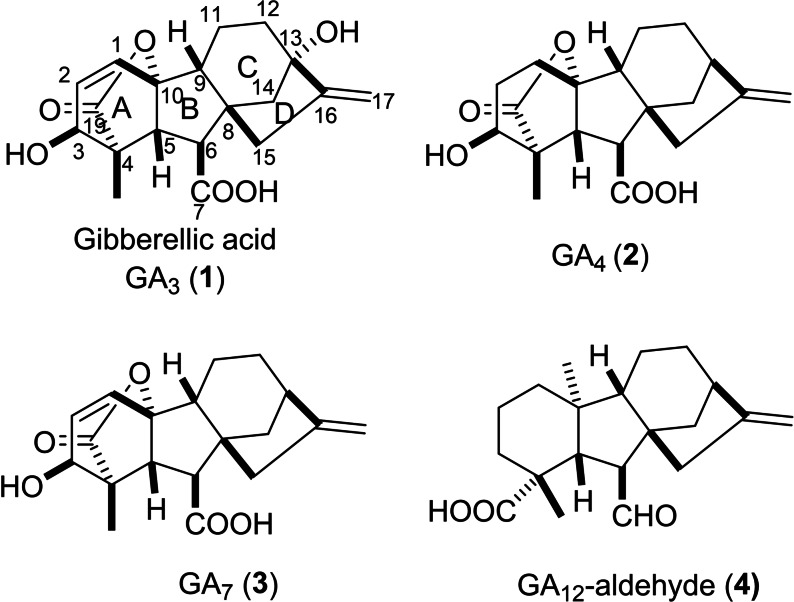
Chemical structure of gibberellin derivatives.

The family of gibberellins (GAs), a group of phytohormones, regulate various stages of plant growth and development,^[1]^ including stem and root expansion,^[2]^ flowering[^3^, ^4^] and seed germination.[^5^, ^6^] The discovery of GAs in Japan in the 1930s can be linked back to the initiation of research on rice infection associated with specific symptoms, such as overgrowth of stems and insufficient seed production. These symptoms are mainly due to exertions of the fungus *Gibberella fujikuroi* (also known as *Fusarium fujikuroi*),[^7^, ^8^] with the main component called gibberellin. Since then, numerous efforts have been employed to elucidate the biosynthetic origin of GA in fungi and later in plants, ultimately converging on geranylgeranyl diphosphate origin with some differences related to regulating enzymes.[^9^, ^10^] In both routes, GA_12_‐aldehyde **4** (Figure 1) is formed and then it is converted to other GAs by the action of a group of specific enzymes.[^11^, ^12^] Afterward, the mechanism of GAs stimulating growth expression was also investigated and could be referred to as inducing DELLA (aspartic acid–glutamic acid–leucine–leucine–alanine) proteins degradation.[^13^, ^14^]

Based on structures and their biosynthetic route, GAs can be classified into two major groups: full diterpenoid skeleton compounds (C_20_‐GAs) and others with 19 carbon atoms (C_19_‐GAs such as GA_3_ **1**, GA_4_ **2**, and GA_7_ **3**) (see Figure 1) that response to main activities.^15^ Gibberellic acid (GA_3_, **1**) is a tetracyclic dihydroxy lactone acid with a perhydrofluorene carbon skeleton with a diversity of functional groups (C_1_−C_2_ double bond, C_10_ γ‐lactone ring and C_13_‐OH).^[16]^ In 1996 Perez *et al*. suggested the key role of C_10_ *γ*‐lactone ring in its biological activity, whereas the stereochemistry of its functional groups has been shown to be responsible for its instability under different conditions.[^16^, ^17^]

### 1.2 GA_3_ Commercial Production

Since the discovery of GA_3_, many reports have highlighted its agro‐industrial applications such as seed germination,[^5^, ^18^] stem elongation,^[2]^ flowering,^[19]^ and inclining fruit growth.^[20]^ Based on these critical activities, many time‐ and cost‐saving and highly productive methods have been developed to produce it in commercial quantities. Among them, fermentation methods including solid‐state fermentation (SSF),^[21]^ semi‐solid‐state fermentation (SSSF)^[22]^ and submerged fermentation (SmF),^[23]^ have been applied. The highest yield of GA_3_ was obtained by Oliveira *et al*. in 2017 using SSF and SSSF methods.^[24]^ These methods, in particular SSF, allow the reuse of agricultural and industrial by‐products, that can be used as substrates for fermentation. As shown in Table 1, a wide range of substrates were employed to enhance the fermentation yields.

**Table 1:** Gibberellic acid production by fermentation using different substrates.

Microorganism	Method	Substrate	Yield
*Fusarium moniliforme*	SSF	Citric pulp	5.9 g/kg^25^
*Fusarium fujikuroi*	SSSF	Citric pulp	4.8 g/kg^24^
*Fusarium fujikuroi*	SSF	Coffee husk	492 mg/kg^26^
*Fusarium fujikuroi*	SSF	Wheat bran	6.8 g/kg^27^
*Fusarium proliferatum*	SSF	Pigeon pea pods	7.8 mg/g^28^

Besides agricultural applications, the structural diversity of GA_3_ has been attracting the attention of synthetic organic chemists not only from the viewpoint of target‐oriented synthesis, but also from the viewpoint of utilizing a new synthetic methodology developed by themselves. This review article focused on the stereoselective syntheses of agents with remarkable pharmaceutical potential, starting from GA_3_ (**1**).

## 2 Ring‐Distortion Strategy of GA_3_

### 2.1 Ring A and Carboxylic Group

**Figure 2:**
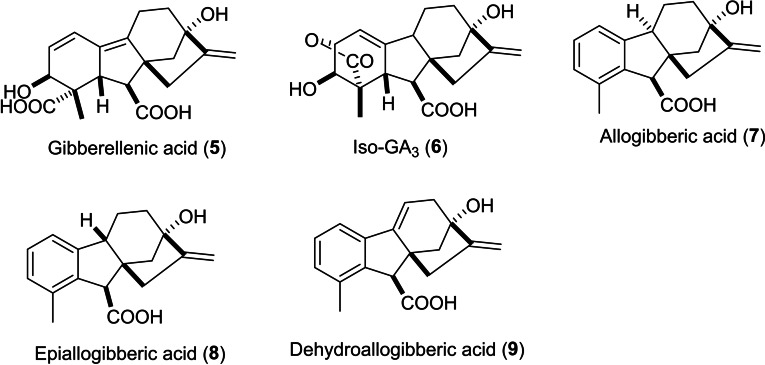
Products of gibberellic acid aqueous decomposition.

The wide diversity of GA_3_ functional groups has contributed to its high reactivity and low stability in both acidic and alkaline conditions. The main stereochemical aspect that facilitates the lactone elimination reaction is the oxygen end of the γ‐lactone ring as it is in allylic position to the C_1_– alkene moiety in a *trans*‐anti‐periplanar orientation concerning the tertiary hydrogen atom at C_9_. Subsequently, GA_3_ goes through lactone elimination, yielding gibberellenic acid even under neutral conditions.[^29^, ^30^] In general, GA_3_ usually breaks down in aqueous solutions into gibberellenic acid **5**, isogibberellic acid **6**, allogibberic acid **7**, epiallogibberic acid **8** and dehydroallogibberic acid **9** (shown in Figure 2) in different ratios based on reaction conditions (reaction time, pH and temperature).^[31]^

**Scheme 1:**
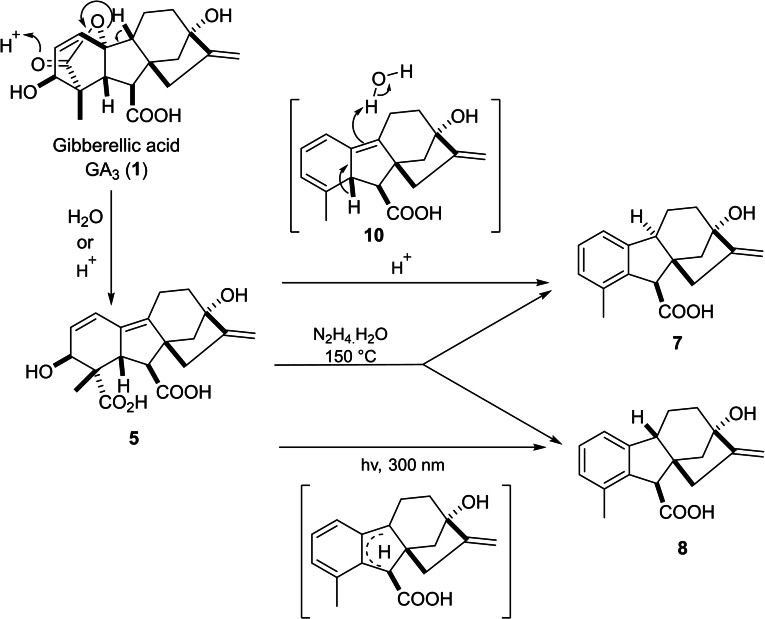
The mechanism of gibberellic acid degradation into epiallo‐ and allogibberic acid.

Pryce suggested the potential mechanism of this decomposition.^[31]^ In this pathway, gibberellenic acid **5** goes through thermal decomposition into intermediate triene **10**, which later yields the major product 9*α*‐H allogibberic acid **7** (with C_9_‐C_10_ *trans*‐fused ring system)^[32]^ through a rearrangement based on solvent exchange. As the C_9_‐C_10_ *cis*‐fused ring system was critical for the synthesis of pharbinilic acid, the preparation of 9*β*‐H epiallogibberic **8** ^[33]^ was carried out by either photochemical transformation of the triene^[34]^ or heating GA_3_ in the presence of hydrazine (Scheme 1).^[35]^

### 2.2 Rings C and D

Various rearrangement reactions of rings C and D in GA_3_ were reported and could be explained by the electrophilic attack on the C_16_‐C_17_ alkene moiety and the presence of the allylic C_13_ hydroxy group.^[36]^

**Scheme 2:**
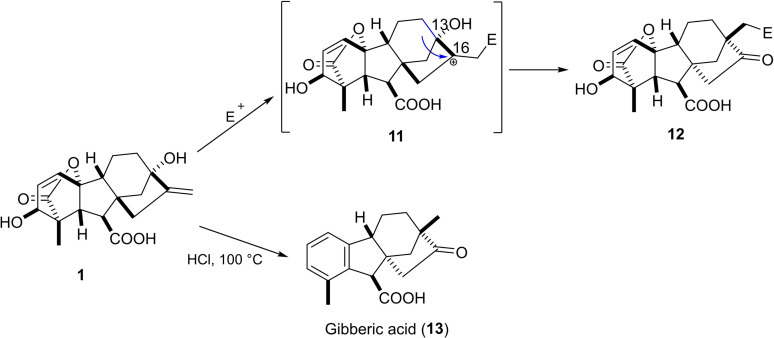
Electrophilic attack and hydroxy‐assisted Wagner–Merwein rearrangement.

Treatment of either GA_3_ or allogibberic acid with concentrated HCl under reflux led to the formation of ketone derivative **13** (gibberic acid).^[37]^ Under these conditions, Wagner–Meerwein rearrangement took place, including the migration of C_12_‐C_13_ to C_16_ with the inversion of the configuration of the C/D rings (Scheme 2).^[38]^

**Scheme 3:**
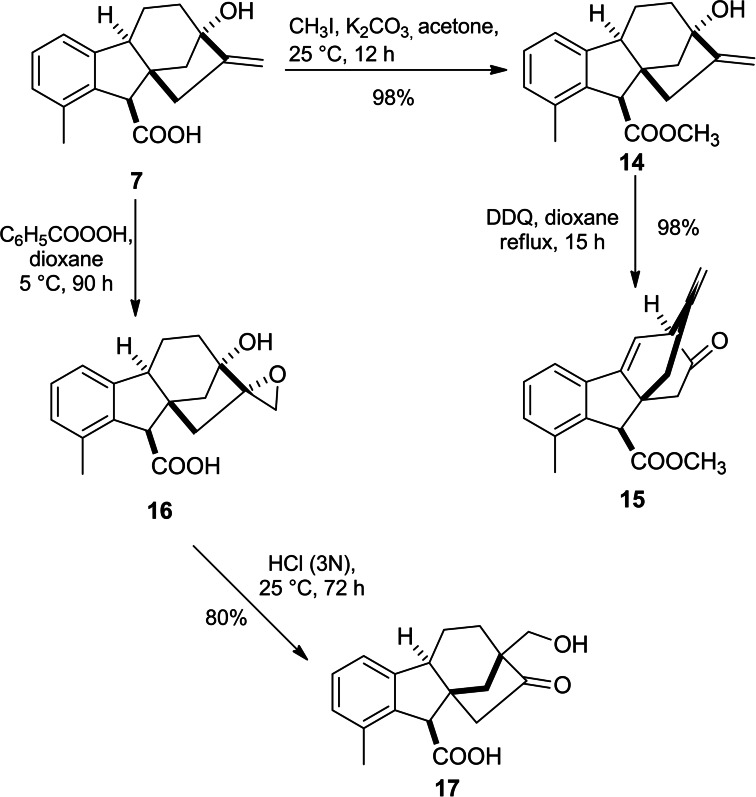
DDQ‐mediated oxidative rearrangement of methyl allogibberate and HCl‐mediated rearrangement of epoxide **16**.

In the presence of DDQ, an oxidative rearrangement of C and D rings of methyl allogibberate yielded α‐keto‐ester **15** due to C_13_‐C_16_ migration to C_12_ (Scheme 3).^[39]^

Hydroxymethyl ketone derivative **17** was successfully obtained through HCl‐mediated rearrangement of epoxide **16**. In detail, this rearrangement took place with the involvement of a 6‐membered transition state followed by Wagner–Meerwein rearrangement to stabilize the carbonium ion (Scheme 3).^[40]^

## 3 GA_3_‐Derived Pharmaceutical Agents

### 3.1 Toxicity Studies

As a consequence of the economic and vital applications of GA_3_ in the agriculture and industrial sectors, studies regarding its potential effects on mammalian systems have emerged. Kimura *et al*. intensely studied the subacute and subchronic toxicity of GA_3_.^[41]^ In both cases, it was indicated that GA_3_ was asymptomatic and it did not induce any histological changes, where the highest tolerable dose in mice was 15 g/kg orally as defined by acute toxicity. Celik *et al*. investigated the potential changes in the antioxidant defence system in rats induced by GA_3_.^[42]^ A high lipid peroxidation rate was detected in specific tissues accompanied by systematic toxicity in the spleen, stomach, heart, lungs and kidneys. Another analysis on the sub‐chronic toxicity of GA_3_ on hepatic functions in adult male albino rats was conducted by Hussein *et al*.^[43]^ A remarkable hepatotoxicity was observed and could be explained by GA_3_‐mediated lipid peroxidation, which increased the MDA levels and thus induced apoptosis and Bcl‐2 overexpression. Troudi *et al*. also reported the potential neurotoxic effects of GA_3_ on pregnant rats in the advanced stages (daily 200 ppm GA_3_ in drinking water).^[44]^ A significant neurotoxicity was observed and manifested by GA_3_‐induced blockage of cerebral and cerebellar AChE and enhanced lipid peroxidation.

### 3.2 Anticancer Activity

Since *ent*‐kaurene diterpenes are well‐known for their critical and various bioactivities including anticancer, antimicrobial and antifungal properties, many efforts have been employed to find GA_3_‐based derivatives with promising biological activities. These can be considered as lead compounds for medicinal chemistry. An investigation accomplished by Koehler in 2009 indicated that Gibberellic acid **1** and 9*α*‐H allogibberic acid **7** could modulate NF‐κB pathway activity and, consequently, they could be utilized for the therapy of several NF‐κB‐related diseases, for instance, cancer and autoimmune diseases.^[45]^

**Scheme 4:**
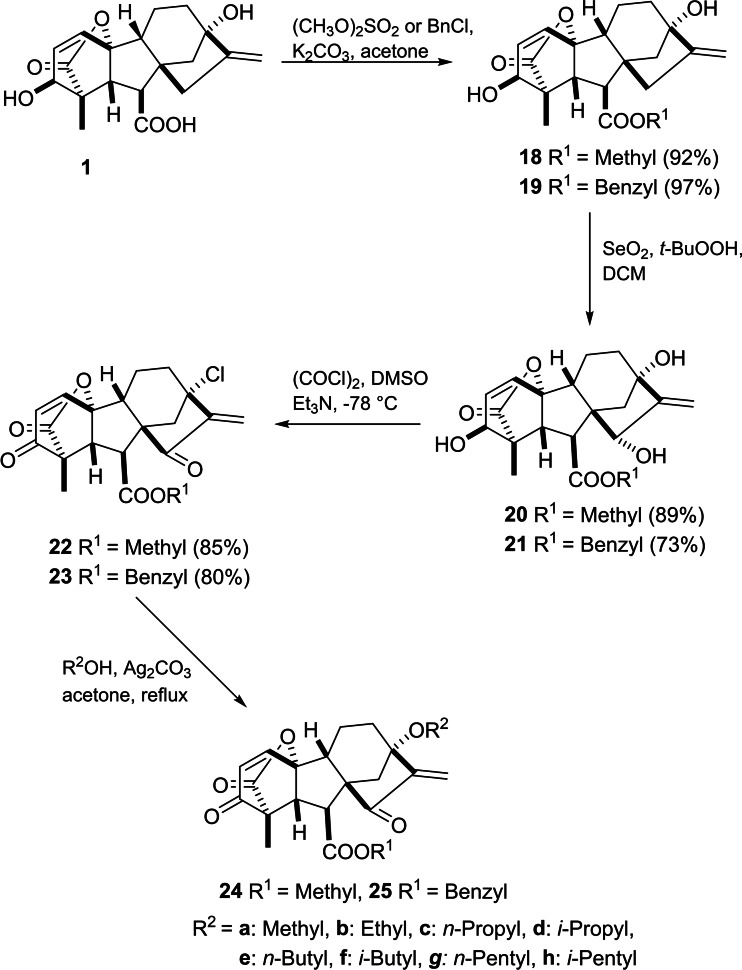
Chemical synthesis of GA_3_‐based diketone derivatives with C_13_ ethereal moiety.

Chen *et al*. introduced a synthetic route to GA_3_‐based *α*,*β*‐unsaturated diketone derivatives (Scheme 4).^[46]^ In this case, the SeO_2_‐mediated allylic oxidation of both the corresponding methyl or benzyl ester **18** and **19** leads to the formation of diols **20** and **21**, respectively. Oxidation of the secondary alcohol group in diols **20** and **21** with oxalyl chloride in the presence of triethylamine in DMSO (Swern oxidation) together with an exchange of the tertiary hydroxy group at C_13_ to chlorine was successfully performed to yield compounds **22** and **23**, respectively. All designed compounds were evaluated against a panel of human cancer cell lines (HT29, A549, HepG2 and MKN28). MTT assay revealed that compounds **22** and **23** exhibited the most significant activity against human colon carcinoma cell line HT29 (IC_50_=2.9 and 4.5 μM, respectively). In the case of human gastric carcinoma cell line MKN28, compound **25a** was found to have the most potent inhibitory activity with an IC_50_ value of 0.8 μM. Moreover, compound **22** showed a complete inhibition towards topoisomerase I at 8 μg mL^−1^ concentration. Furthermore, as reported by Zhang *et al*., the *in vitro* and *in vivo* antiangiogenic activities of compound **22** downregulated VEGF receptor signaling.^[47]^

Following this procedure, an additional structural modification was reported by Jingbo *et al*. to obtain derivatives bearing an ethereal moiety at C_13_ (see Scheme 4) by converting the chlorine substituent into a variety of alkoxy groups in the presence of silver carbonate. This structural modification led to compound **25h**, which is an efficient compound against MKN28 with a low IC_50_ value (0.21 μM).^[48]^

**Scheme 5:**
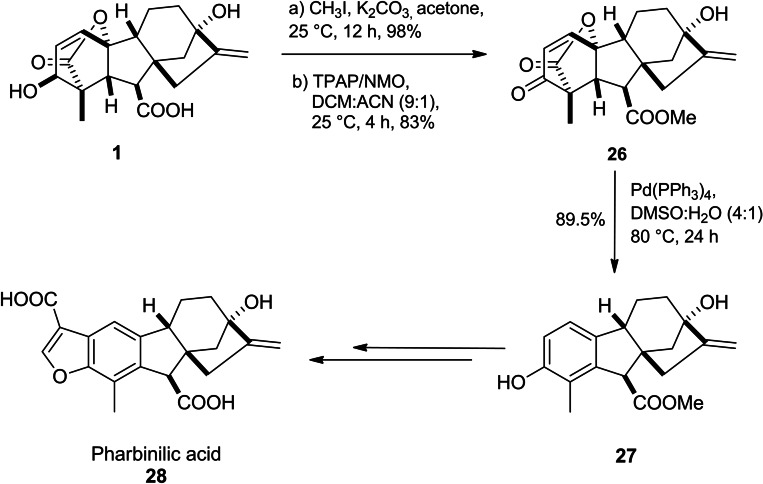
Chemical synthesis of pharbinilic acid.

Pharbinillic acid **28**, an epiallogibberic acid‐related compound, isolated from Pharbitis nil, exhibited cytotoxicity against several cancer cell lines (A549, SK‐OV‐3, SK‐MEL‐2 and HCT‐15) as well as modulated NF‐κB activity. Annand *et al*. reported a seven‐step synthetic pathway of pharbinilic acid starting from GA_3_.^[36]^ The key intermediate, hydroxy epiallogibberic methyl ester **27**, was obtained through Griffith–Ley oxidation (TPAP/NMO) of GA_3_ methyl ester to produce enone **26** and then it was subjected to Pd(PPh_3_)_4_‐mediated ring A aromatization to form target product **28** (Scheme 5). Further investigations have shown that enone **26** selectively inhibited nuclear translocation of IKKα and displayed selective cytotoxicity towards cancerous cell lines.^[49]^

**Scheme 6:**
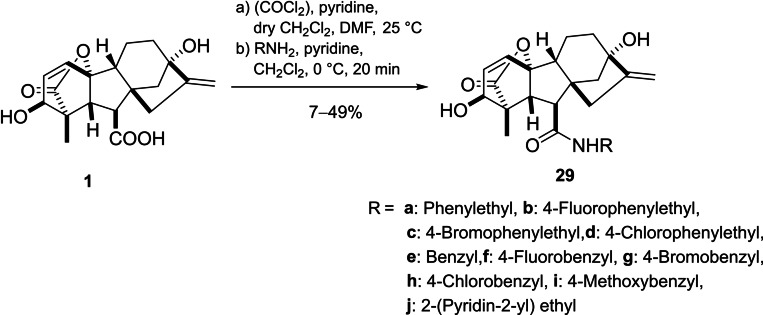
Preparation of gibberellic acid‐based amides.

Egbewande *et al*. reported a parallel‐solution‐phase synthesis of GA_3_ amides (Scheme 6). The carboxylic group was activated by oxalyl chloride to yield the corresponding acyl chlorides, which were then coupled with the desired amines to form compounds **29**. A considerable reduction in free cholesterol uptake in prostate cancer cells was observed upon treatment with compounds **29b‐c**, **29g‐h** and **29j**.^[50]^

**Scheme 7:**
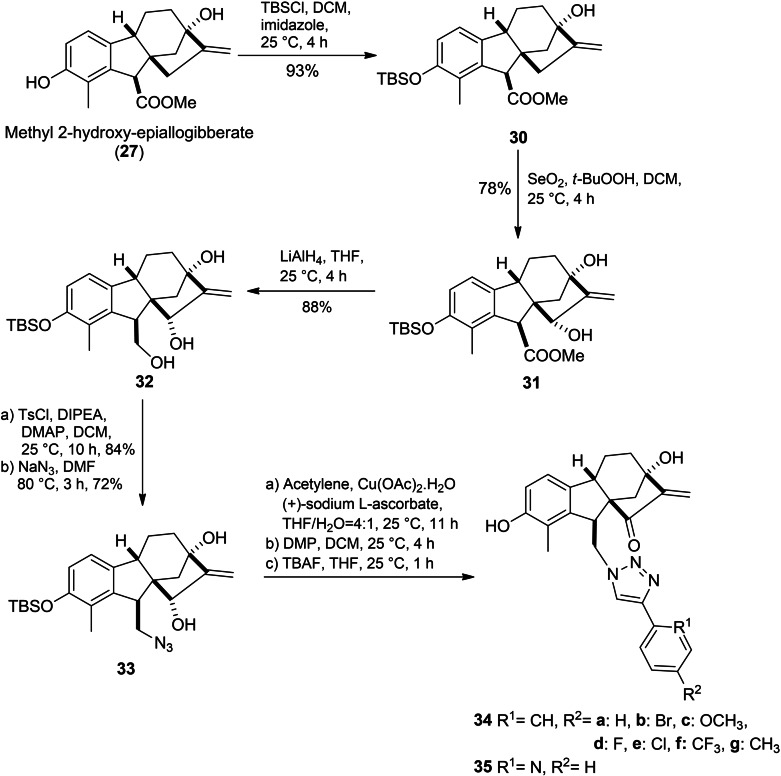
Synthesis route of 1,2,3‐triazoles based on hydroxy epiallogibberil.

A series of hydroxy epiallogibberic acids coupled with 1,2,3‐triazole bearing an *α*,*β*‐unsaturated ketone moiety was synthesized by Wu *et al*. (Scheme 7). In this protocol, methyl hydroxy epiallogibberate **27** was obtained by (Dess–Martin periodinane) DMP‐mediated oxidation of GA_3_ methyl ester. Phenol protection with TBSCl followed by allylic oxidation mediated with SeO_2_/*t*‐BuOOH and subsequent reduction of ester group with lithium aluminium hydride (LAH)‐based yielded compound **32**. Next the primary alcohol group was tosylated and then it was treated with sodium azide to provide azide **33**. The desired triazoles were obtained from compound **33** by a three‐step sequence including CuAAC azide–alkyne cycloaddition, Dess–Martin oxidation and deprotection. The *in‐vitro* evaluation confirmed the essential role of the *α*,*β*‐unsaturated ketone moiety in cytotoxicity against cancer cell lines, where compounds **34c**, **34d** and **34e** exhibited potent cytotoxicity, which is 8 to 20 times stronger than that of reference cisplatin. Compound **34d** was found to trigger cell cycle arrest in the S phase and promoted apoptosis in SMMC‐7721 cell lines.^[51]^

**Scheme 8:**
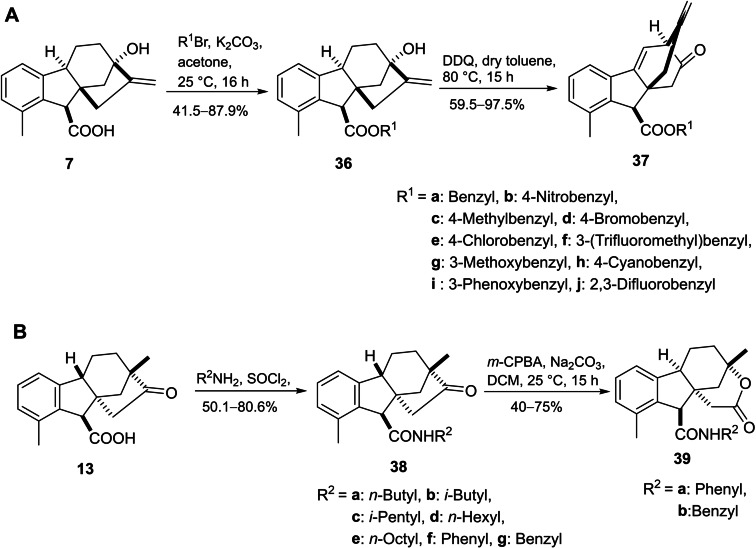
Synthesis of allogibberic acid‐derived ester (**A**) and amide derivatives (**B**).

In order to further investigate potential GA_3_‐based antitumor derivatives, Zhu *et al*. designed and synthesized a library of allogibberic acid‐substituted benzyl esters **36** and their keto derivatives **37** through DDQ‐mediated rearrangement of the corresponding ester (Scheme 8A).

On the other hand, a series of gibberic carboxamides was also prepared by treatment of amides **38** under Baeyer–Villiger oxidation conditions (*m‐*CPBA) leading to the formation of corresponding lactones **39** (Scheme 8B). The cytotoxicity of the designed compounds varied among the cancer cell lines. Within the ester derivatives, compounds featuring the *meta*‐substituted benzyl group displayed greater activity than those with a *para*‐substituted benzyl substituent with more selectivity towards HL‐60. Considering the amides, compounds with linear saturated substituents showed the best activity, especially compound **38e**, which had strong activity against all tested cell lines (HL‐60, MCF‐7, SW480 and NCI−H226).^[52]^

**Figure 3:**
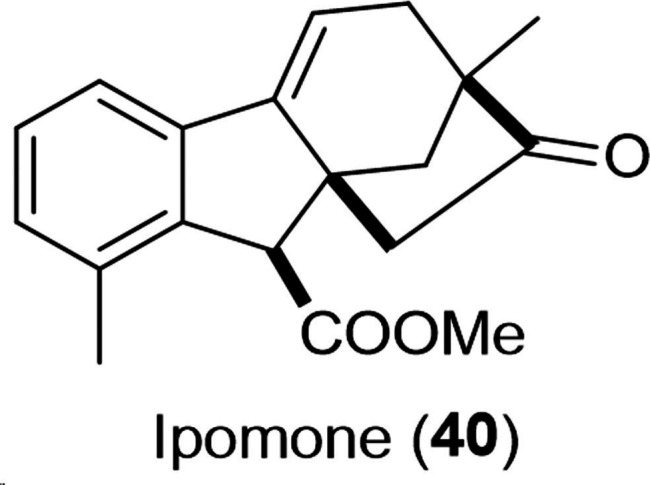
Chemical structure of ipomone **40**.

Recently, a new gibberic acid derivative, the methyl ester of dehydrogibberic acid (ipomone **40**, shown in Figure 3), was successfully isolated and identified by Goel *et al*. from acidified hydroalcoholic extract of *Ipomoea nil* seeds.^[53]^ The suggested mechanism of ipomone formation was the acid‐mediated rearrangement of dehydroallogibberic acid. Despite the low cytotoxicity of ipomone against cancer cell lines (IC_50_=34–86 μM), the western blot analysis indicated a dose‐dependent decline in the expression of caspase‐3 and PARP‐1 in lung cancer A549 cells approving that ipomone could stimulate apoptosis and autophagy in these cells.

**Scheme 9:**
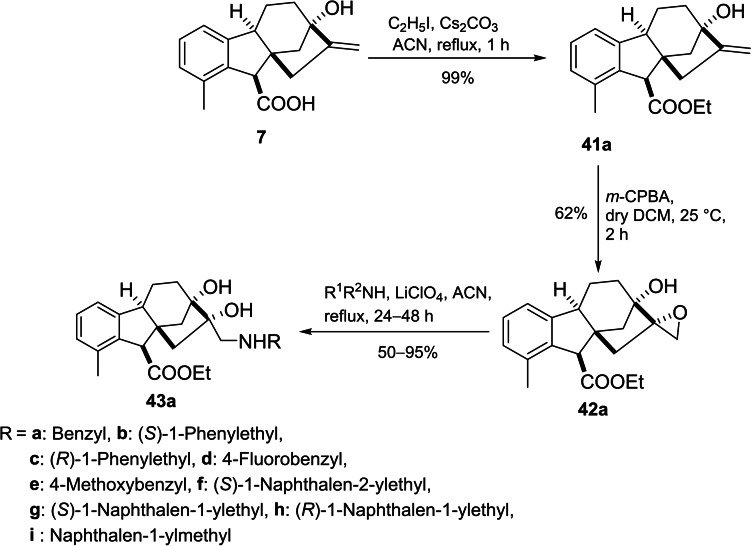
Preparation of aminodiols **43a** derived from allogibberic acid.

Khdar *et al*. introduced the synthetic route of a library of aminodiols based on allogibberic acid (Scheme 9).^[54]^ The methodology involved the esterification of allogibberic acid followed by stereospecific synthesis of epoxide and subsequent ring opening with various primary and secondary amines catalyzed by lithium perchlorate to produce aminodiols **43a**.

**Scheme 10:**
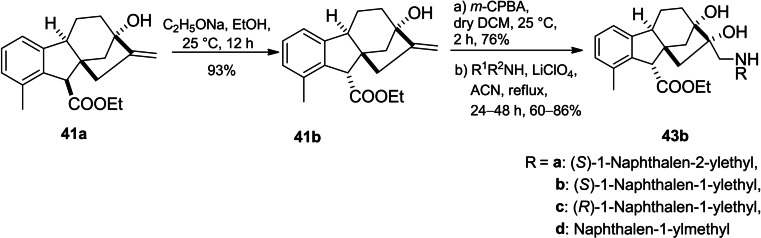
Preparation of aminodiols **43b** derived from allogibberic acid.

Considering another possibility, the isomerization of the ester group of **41a** under alkaline conditions followed by epoxidation and then aminolysis of the resulting epoxide led to the formation of aminodiols **43b** (Scheme 10).

The MTT assay demonstrated that secondary aminodiols (especially naphtyl ethylamine derivatives with IC_50_=4–7 μM) possessed the most important antiproliferative activity against a panel of cancer cell lines (A2780, HeLa, SiHa and MDA‐MB‐231). In contrast, the highest cancer selectivity was shown by compound **43ag**. Moreover, the importance of the *R* configuration of the ester group for antiproliferative activity was also indicated.^[54]^

**Scheme 11:**
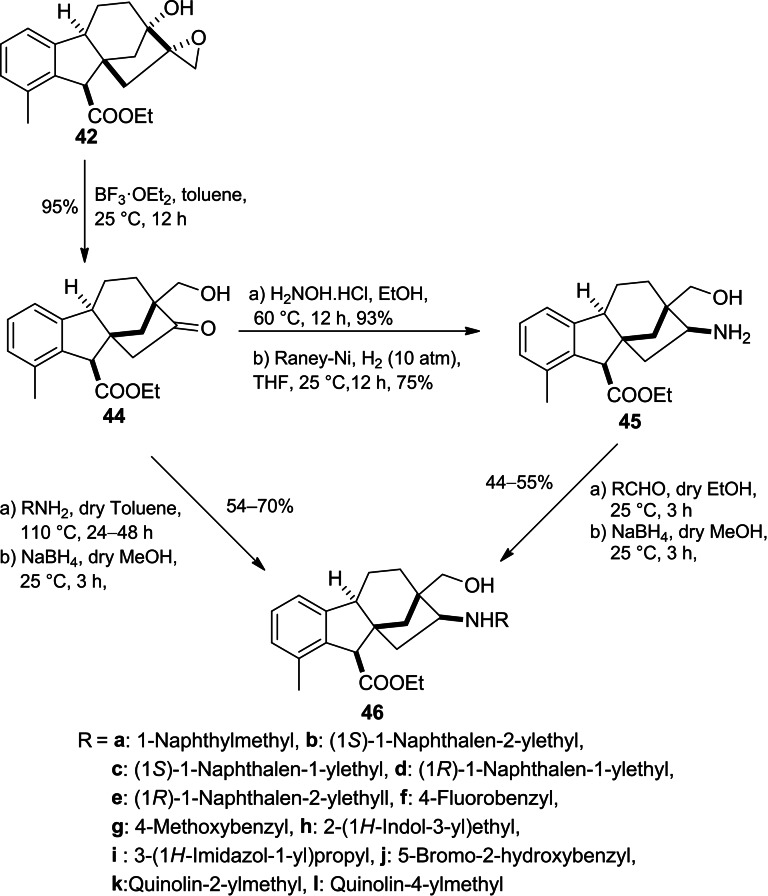
Preparation of aminoalcohols **46** derived from allogibberic acid.

Continued interest in the modification of allogibberic acid, compound **44** was successfully prepared by Khdar *et al*. through BF_3 ⋅_ OEt_2_‐mediated rearrangement of epoxide **42** (Scheme 11).^[55]^ This step produced the groundwork to design a library of 1,3‐aminoalcohols **46** by either reductive amination of the hydroxymethyl ketone **44** or reductive alkylation of primary aminoalcohol **45**.

**Scheme 12:**
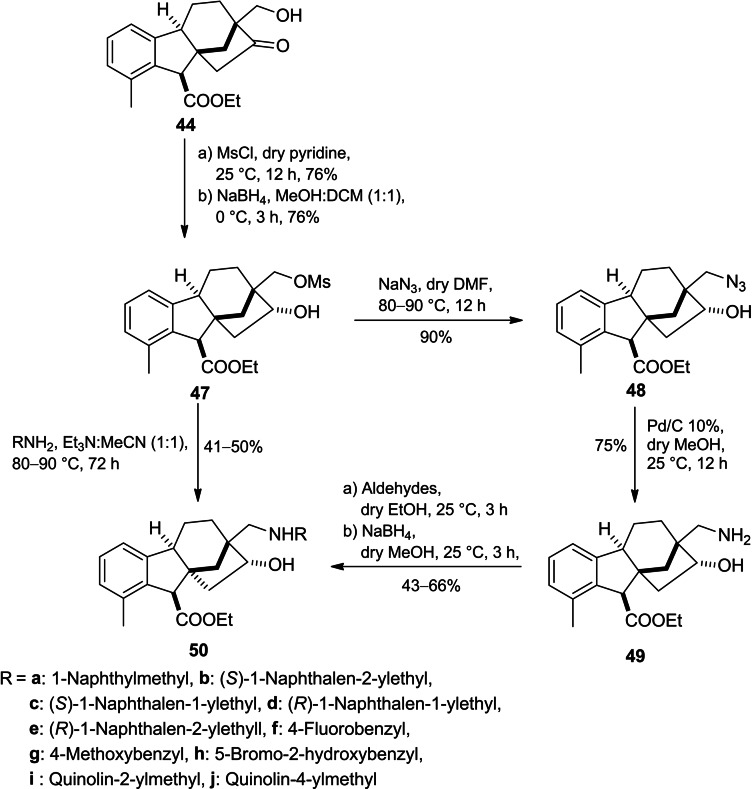
Preparation of aminoalcohol **50** derived from 1,3‐allogibberic acid.

In addition, the corresponding regioisomers **49** were successfully synthesized in three steps including reduction of mesyl derivative **44** followed by azidation of the corresponding aldohol **47** and subsequent Pd‐catalysed hydrogenolysis of azide derivative **48**. Furthermore, the exchange of the mesyl function in mesylate **47** with various primary amines or reductive alkylation of primary aminoalcohol **49** yielded the desired 1,3‐aminoalcohols **50** (Scheme 12).

According to the MTT assay, a marked and substantial variation was discerned in the antiproliferative activity exhibited by the regioisomers. Substituted benzyl derivatives (**50f**, **50g** and **50h**) and indolyl derivative **46h** showed potent activity with a cell line‐dependent effect. Moreover, they also displayed modest cancer selectivity with calculated IC_50_ values higher on NIH/3T3 fibroblast cells compared to tested cancer cell lines.

**Figure 4:**
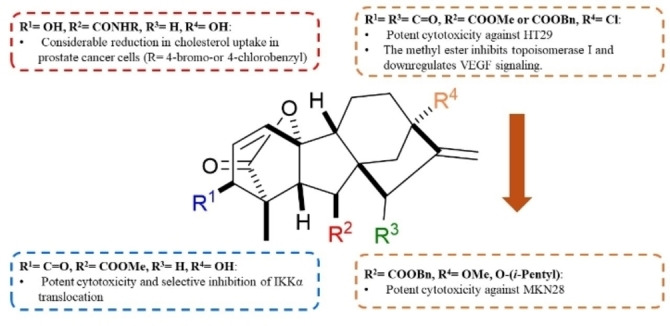
Structure‐activity relationship of anticancer activity of gibberic acid.

**Figure 5:**
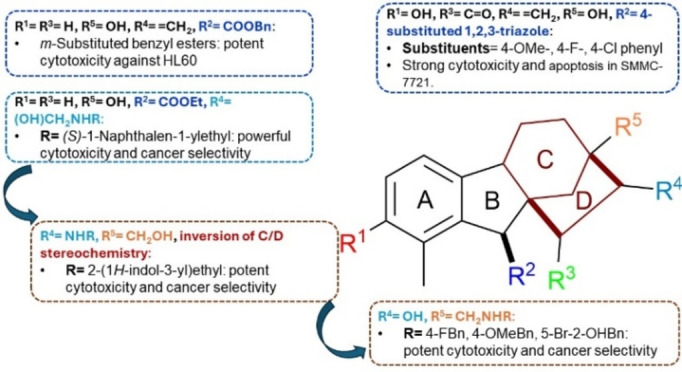
Structure‐activity relationship of anticancer activity of allogibberellic acid.

Based on the aforementioned structural modifications, the structural aspects responsible for the anticancer activity of gibberellic acid and allogibberic acid was summarized as shown in Figures 4 and 5. It is obvious that the presence of diketone at positions 3 and 15 of methyl or benzyl gibberrate along with the substitution of OH at position 13 with Cl, OMe, or O‐(*i*‐pentyl) is critical for promising anticancer activity. The combination of ring A aromatization and benzyl ester functionality gave rise to new promising derivatives. The introduction of the OH group at position 2 on the aromatic ring A and a ketone group at position 15, as well as the transformation of the carboxyl group into 4‐substituted 1,2,3‐triazole, showed a crucial role in the activity.

On the other hand, bearing ethyl allogibberate with 3‐amino‐1,2‐diol motif or with 1,3‐aminoalcohl moiety alongside inversion of rings C/D stereochemistry also displayed a significant influence on the activity.

### 3.3 Antimicrobial Activity

**Figure 6:**
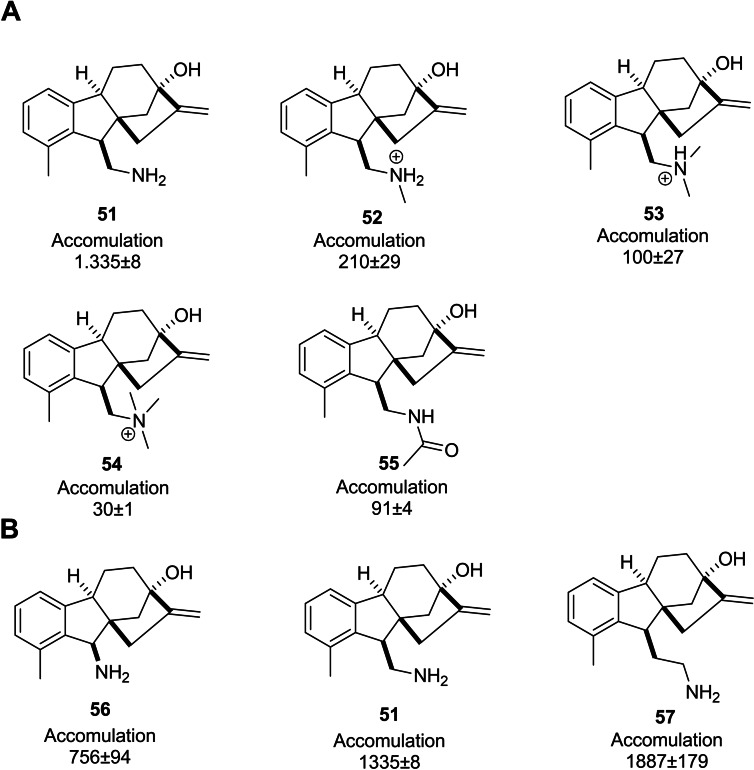
The *E. coli* accumulation of several amines derived from allogibberic acid.

Richter *et al*. conducted a comprehensive investigation into the accumulation of a wide variety of small molecules in Gram‐negative bacteria, especially *E. coli*, aiming at highlighting the potential factors that affect accumulating compounds. This study established the high accumulation of primary amine derivatives of allogibberic acid in *E. coli* MG1655. Its monomethyl, dimethyl, trimethyl, and acetylated derivatives (Figure 6A), in turn, exhibited a dramatic decrease in accumulation. It was also concluded that when the primary amine functionality was more systematically separated from the condensed ring system, the compounds had more accumulation (Figure 6B).^[56]^

**Figure 7:**
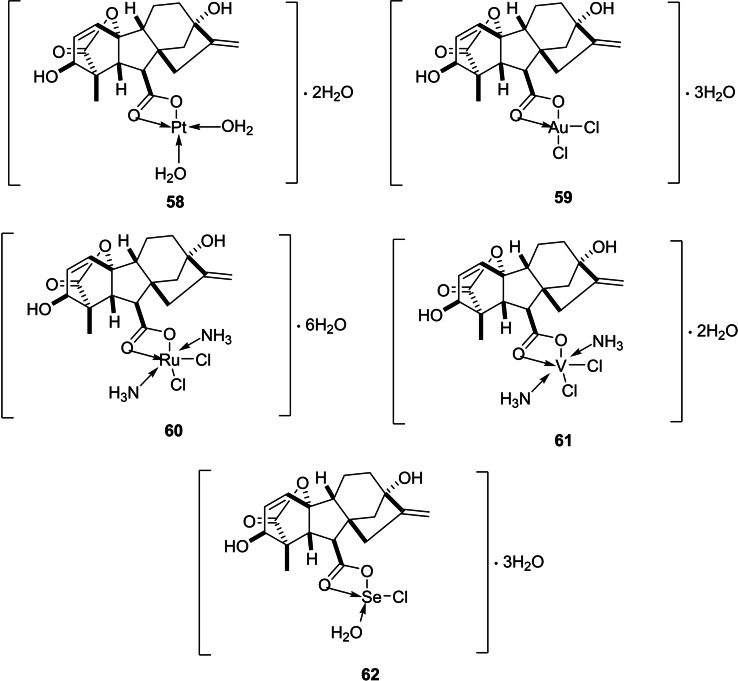
Chemical structure of complexes with several ions derived from gibberellic acid.

Another study was undertaken by El‐Sayed *et al*. with the objective of preparing and characterizing a group of GA_3_ complexes with Pt(II), Au(III), Ru(III), V(III), and Se(IV) ions (Figure 7). The study included biological evaluation as well. Reactions were conducted under neutral pH, in which the anionic form GA_3_^−^ was dominant. The desired complexes were obtained by the reaction of the ions with GA_3_^−^ at 65 °C. The Kirby‐Bauer disc diffusion method indicated that Au(III) and Se(IV) complexes displayed integral antimicrobial activity, particularly against *E. coli* and *S. aureus*. The cytotoxicity of GA_3_ against HepG2 and MCF‐7 cell lines, in turn, was remarkably increased when it was combined with Pt(II) and Au(III) ions.^[57]^

In 2021, Toner *et al*. conducted an investigation on the antimicrobial effects of several diterpene phytohormones (gibberellins) against a group of phytopathogens and clinical pathogens.^[58]^ The obtained results showed that GA_3_ **1** failed to inhibit the growth of bacterial organisms of clinical significance to humans, while GA_4_ **2**, a gibberellin lacking C_1_−C_2_ double bond, and C_13_−OH, displayed a strong inhibition against all the tested clinical bacteria including Gram‐negative and Gram‐positive ones.

These results emphasize the importance of both the transformation of the carboxylic group into primary amine functionality and the formation of carboxylate complexes with several ions for antimicrobial activity.

### 3.4 Anti‐Inflammatory Activity

Since the ability of gibberellic acid **1** to modulate NF‐κB pathway activity was declared by Koehler in 2009,^[45]^ the possible use of gibberellic acid to modulate inflammation response has been studied. Commencing from the standpoint that gibberellic acid can trigger A20‐like zinc finger proteins in plants,^[59]^ Reihill *et al*. investigated the A20‐mediated anti‐inflammatory effect of gibberellic acid on airway epithelial cells.^[60]^ The results showed that the higher levels of A20 mRNA and protein together with a considerable decrease in IL‐6 and IL‐8 release were observed in epithelial cells treated with gibberellic acid. This effect was regulated by GA_3_‐mediated induction of zinc finger protein A20 resulting in an incline in 1κBα levels and consequent decrease in NF‐κB expression. These findings suggest the possible use of gibberellic acid for chronic inflammation treatment. Recently, Xu et al. reported the role of gibberellic acid in reducing sepsis‐based neuroinflammation.^[61]^ It was found that the incubation of microglial cells (the primary immune cells in the brain) with gibberellic acid resulted in overexpression of ZBTB16, an NF‐κB regulator, diminishing the LPS (Lipopolysaccharide)‐stimulated microglial M1 activation releasing anti‐inflammatory activity.

These investigations provide a strong foundation for the development of GA_3_‐based derivatives with promising anti‐inflammatory activity.

### 3.5 Other Activities

Besides the previously mentioned interesting bioactivities of gibberellic acid, other biological properties were reported in the literature. Kasamatsu *et al*. declared the successful use of gibberellic acid for the regeneration of salivary glands starting from adipose‐derived stem cells, where high levels of α‐amylase were detected in stem cells after 7 days of occupation with gibberellic acid.^[62]^

On the other hand, the gibberellic acid hormonal effects on plants, including stem and root elongation and enhancement of flowering and fruit production, were extensively studied in the literature. The main mechanism that accounts for its actions is inducing DELLA protein degradation by binding GA_3_ with the DELLA receptor leading to conformational changes and subsequent degradation.[^8^, ^19^, ^63^, ^64^, ^65^, ^66^]

## 4 Summary and Outlook

In conclusion, this review provides insights into the structural features underlying the potential biological activities of gibberellic acid and its derivatives, shedding light on the most promising derivatives with anticancer activity. Numerous structural modifications have been reported and a wide range of compounds bearing antiproliferative activity with diverse potencies and selectivities was reported. By critically evaluating existing pieces of information in the literature, it is proven that several functionalities, for instance, *α*,*β*‐unsaturated ketone, 1,2,3‐triazoles, *meta*‐substituted benzyl derivatives, amides, aminoalcohols and aminodiols, are crucial to the anticancer activity.

Future research should explore interdisciplinary methods to further elucidate the possible mechanisms of actions and potential targets. This could lead to more efficient structural modifications based on compound/target interactions. Moreover, a combination of the aforementioned structural modifications including aminodiols or aminoalcohols of meta‐substituted benzyl allogibberic acid esters, derivatives with substituted ring A and aminoalcohols, aminodiols or triazoles of pharbanillic acid, will allow additional evaluations. In the meantime, these derivatives should also be scanned for further biological activities (antifungal, antimicrobial and anti‐inflammatory activities).

What might the future application of GA_3_ look like? Owing to their low cost and structural diversity, GA_3_ could be extensively utilized by synthetic chemists in the total synthesis of natural products.^[67]^ The functional groups make a variety of stereoselective transformations of GA_3_ possible, such as acylation or alkylation at the *α*‐position, while the double bond can be transformed by catalytic reduction, epoxidation, dihydroxylation, oxidative cleavage and ring contraction, to name a few possibilities.^[68]^ In addition, the *ent*‐kaurene framework, having multiple asymmetric centres, will certainly play an important role as a promising starting material for natural product syntheses in the future.^[69]^

## Author Contributions

Z.A.K.: Writing – original draft preparation; T.M.L.: writing – review and editing; Z.S: supervision and editing, funding acquisition. All authors have read and agreed to the published version of the manuscript.

## Conflict of Interests

The authors declare no conflict of interest.

5

## Biographical Information

*Zein Alabdeen Khdar was born in Latakia, Syria in 1990. He received his bachelor's degree in pharmacy and pharmaceutical chemistry at Tishreen University, Syria in 2013. Then he obtained his MSc degree in drug design and quality control at Tishreen University in 2019. Since 2021, he has started his PhD in Pharmaceutical Sciences at the University of Szeged, Hungary. His research interests vary from the stereoselective synthesis of diterpene‐based aminoalcohols, aminodiols, and aminotriols to the In‐silico studies, including docking and ADMET analysis*.

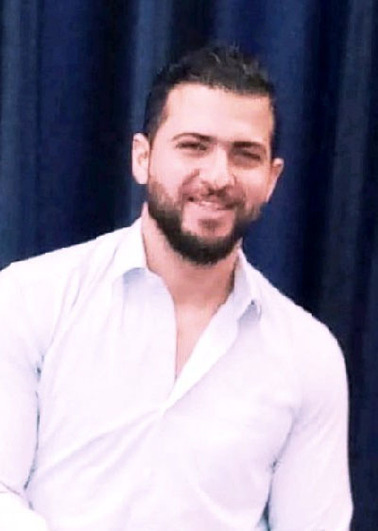

## Biographical Information

*Tam Minh Le was born in Vietnam in 1989. He received his MSc degree in Chemistry in Vietnam (2015) and his PhD in Pharmaceutical Science at the University of Szeged, Hungary (2020), where he continues to his research as a member of Hungarian Academy of Sciences in Institute of Pharmaceutical Chemistry until now. His research interests range from stereoselective synthesis of monoterpene‐based amino acid derivatives to the transformation of natural products, especially in the terpenoid field*.

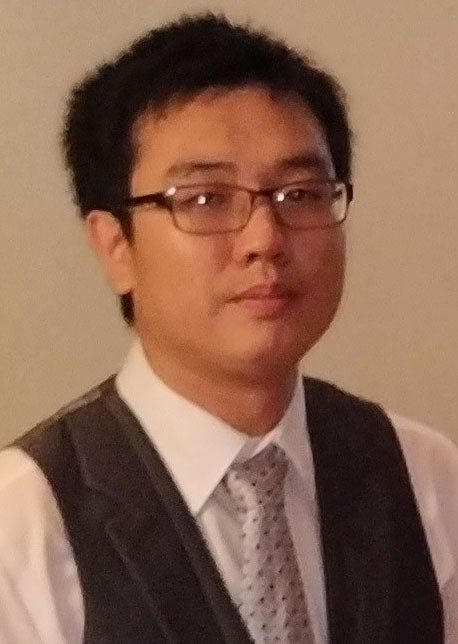

## Biographical Information

*Zsolt Szakonyi was born in 1969 in Szombathely, Hungary. He received his Master's degree in Pharmacy in 1992. He undertook his PhD in synthetic organic chemistry in the group of Prof. Ferenc Fülöp and held his Ph.D. Degree at the University of Szeged (Hungary) in 1998. He joined the research group of Prof. Norbert De Kimpe at the University of Ghent, Belgium in period of 1999–2000 as a postdoctoral Research Fellow by FWO. He became full professor at the Institute of Pharmaceutical Chemistry, University of Szeged in 2019. His research interests centre on stereoselective synthesis of monoterpene‐ and diterpene‐based bi‐ and tridentate building blocks, beta‐amino acid derivatives, amino diols and saturated 1,3‐heterocycles with a special focus on homogeneous stereoselective catalysis*.

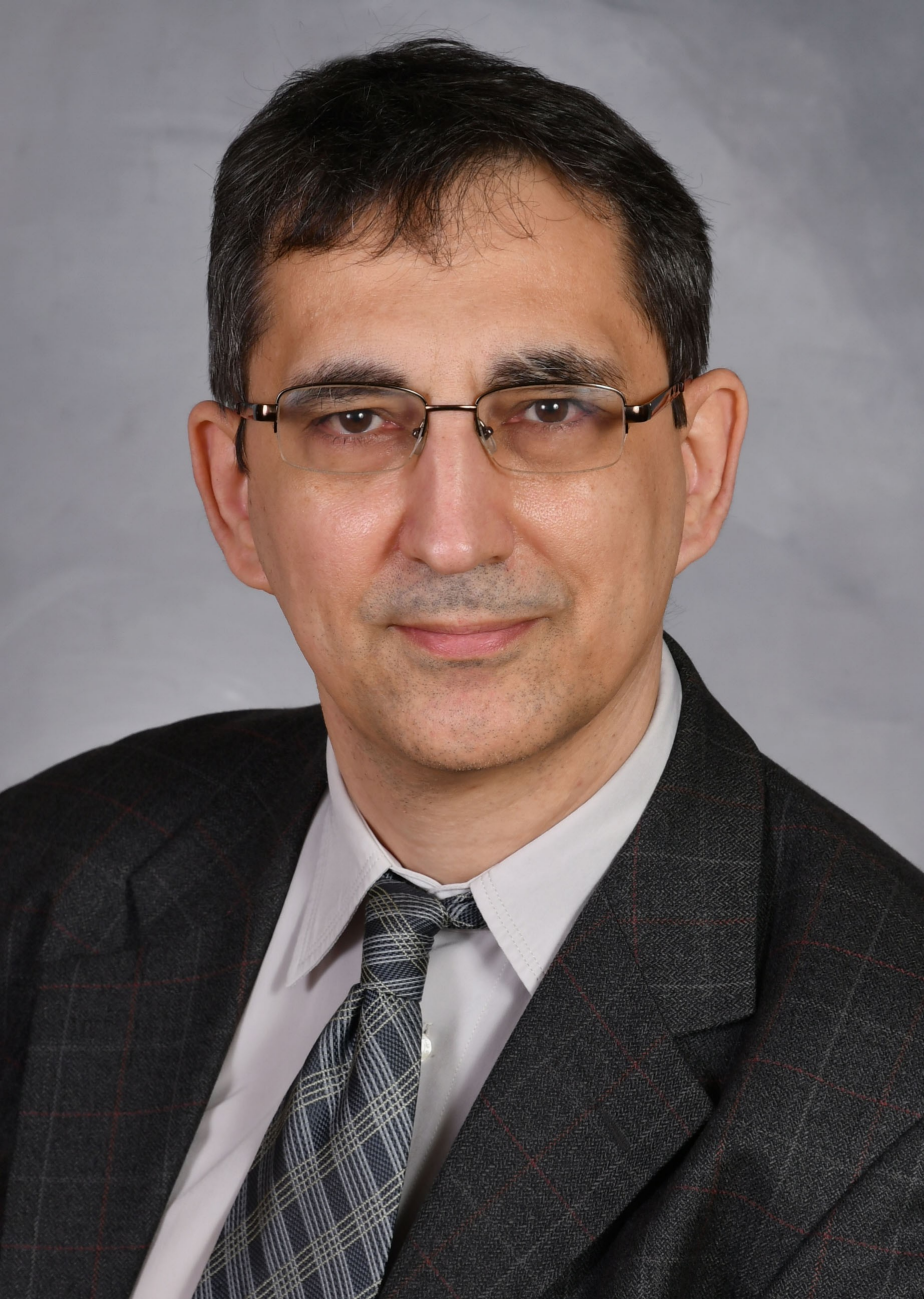

## Data Availability

Data sharing is not applicable to this article as no new data were created or analyzed in this study.
